# Exploring the organisation and delivery of falls management in care homes for older people in England

**DOI:** 10.1186/s12877-025-06127-w

**Published:** 2025-07-01

**Authors:** K. Robinson, P. L. Logan, A. L. Gordon, S. Timmons, T. Masud, L. Rees, A. Kilby, W. Lawry, F. Hallam-Bowles

**Affiliations:** 1 Centre for Rehabilitation and Ageing, University of Nottingham, Nottingham, UK; 2 Research and Innovation, Nottingham University Hospitals NHS Trust, Nottingham, UK; 3 University of Queensland, Brisbane, Australia; 4 Academic Centre for Healthy Ageing, Queen Mary University of London, London, E1 4NS UK; 5 NIHR Applied Research Collaboration- East Midlands (ARC-EM), Nottingham, NG7 2TU UK; 6 Centre for Health Innovation, Leadership and Learning, University of Nottingham, Nottingham, UK; 7 Avery Healthcare Group, Northampton, UK; 8 Nottinghamshire Healthcare NHS Foundation Trust, Nottingham, UK; 9 Royal Devon University Healthcare NHS Foundation Trust, Exeter, UK

**Keywords:** Long-term care, Implementation, Falls prevention

## Abstract

**Background:**

To explore the organisational context of English care homes in delivering falls management and identify barriers and facilitators to help design future service delivery.

**Methods:**

Non-participant observations and semi-structured interviews in one region of England with care home staff, commissioners and individuals involved in the organisation of falls management, care home managers, care home owners and residents. Barriers and facilitators were identified by thematic analysis and mapped against the Consolidated Framework for Implementation Research (CFIR).

**Results:**

17 interviews were undertaken including staff and a resident from three care homes. Delivering falls management in care homes was complex and challenged by difficulties in integration across a disjointed system, workforce challenges and managing complexity of resident needs with multiple competing priorities. Facilitators included consistent and regular multi-disciplinary support, valuing team working within the care home, and between the care home and external agencies, and the ability to retain care home staff who developed and honed skills over time and who valued their advocacy role for residents. Variation in care home environments, and access to healthcare support were highlighted.

**Conclusions:**

The delivery of falls management in care homes is complex and involves a number of interacting systems. Implementation strategies to support future delivery need to consider the pressure on care homes and wider systems, workforce challenges and variation between settings.

**Supplementary Information:**

The online version contains supplementary material available at 10.1186/s12877-025-06127-w.

## Background

Long term care homes provide round the clock personal care, which may be with or without nursing provision, to residents who may have disability or dependency including frailty, dementia, or complex social and medical needs. Care home is an umbrella term to cover both residential and nursing homes in the UK [1]. Care homes in the UK vary in their organisational characteristics, such as their size, ownership and environment [2, 3]. Care home residents are at high risk of falling [4]. Falls can lead to serious injuries, hospital admissions and reduce independence, confidence and quality of life. As such the prevention and management of falls for care home residents is a priority for health and social care within national policy guidance [5]. The World Falls Guidelines recommend all care home residents should be considered at risk of falling, with a multifactorial assessment undertaken to identify actions to reduce risks [6]. A systematic review with intervention component analysis has identified that for multifactorial interventions to be effective they need to be tailored to individual residents, be co-designed with the care home sector and engage care home staff during implementation [7]. One such programme is the Action Falls programme.

The Action Falls programme is a multi-domain decision tool designed to identify falls risk factors and strategies to reduce these. The programme which was co-produced by the clinical, research and care home community demonstrated a 43% reduction in falls through a randomised controlled trial in care homes in England [8]. The process evaluation within the trial found challenges in embedding the programme into routine care home practice and suggested that the context of care homes needed to be further understood when considering implementation of interventions [9].

Older people living in care homes internationally, have very similar care needs and levels of functional dependency, however the way in which care is delivered and organised can differ. In England, care homes provide care through collaboration with multiple external statutory and non-statutory health and social care providers. Care home providers are usually outside of the National Health Service (NHS) and are commonly run by private companies [10]. Funding is varied with contributions from individuals, local authorities, and the NHS. Care homes in England are regulated by the Care Quality Commission. The contextual factors which influence care home readiness for change, implementation and sustainability of healthcare interventions in care homes [11], and the conduct of research in care homes [12] have been well described. These include legislative, organisational and cultural issues which vary mostly at the care provider company and individual care home levels. These well documented issues around organisation of care homes also impact on how falls management can be delivered and sustained [13].

Falls prevention is identified as a priority for care homes and Action Falls is the recommended approach in the NHS England Enhanced Health in Care Homes framework [5]. However there is no mandate for Integrated Care Systems or care provider organisations to adopt this recommendation at a regional or local level and this has led to implementation of a variety of falls prevention practices.

Collaboration with care homes is more established in some areas than others and different models of support are being trialled, resulting in contextual differences [14]. Further, quality of care within care home organisations is likely to be influenced by a complex interaction of factors, including managerial stability, leadership and organisational values, staffing and autonomy [15]. Thus, the organisation of care homes and delivery of care is similarly expected to impact on how falls management programmes can be delivered and sustained. To explore how Action Falls can be successfully embedded into routine care practice, exploration of the context of care homes is needed.

This study aimed to explore the organisational context of English care homes in delivering falls management and identify barriers and facilitators to implementing falls management programme to help design ways to support future delivery.

## Methods

A qualitative study was undertaken with two forms of data collection that occurred concurrently: non-participant observations and semi-structured interviews. The study is reported in line with the Standards for Reporting Qualitative Research (supplementary information file 1) [16].

### Data collection

#### Non-participant observations

Any care home registered to support older people in the East Midlands region in England was eligible to take part. Care homes were approached to take part through the Enabling Research in Care homes network and by identifying information from publicly held databases such as the Care Quality Commission. Care home owner and/or managers were approached with a care home information sheet. From those that agreed to participate, a bespoke agreement was used to confirm the care home were happy for the research to be conducted within the setting. Non-participant observations of care home staff were conducted in three care homes for older people in the East Midlands of England by one researcher who received training on ethnography (FHB). 1940 min of observation were completed over 28 observation periods. All three homes were registered for nursing care and had between 33 and 40 residents. Staffing types included in observations within each care home are included in Table 1. The overarching corporate structure for each home differed between homes, with one part of a chain of two, one a chain of six homes, and one a single home operating as a small independent business. The observations focused on the organisational and contextual factors relating to delivering falls management. This included the observation of multidisciplinary meetings involving external services, handover meetings (meetings to transfer information between care staff about residents usually between shifts), staff meetings and interaction between staff in communal areas of the care home. A flexible collaborative approach was taken to schedule observation visits. Potential observation opportunities were identified with care home managers. Observations were completed over 1–2 months in each care home and the frequency was guided by weekly conversations with the care home manager. The duration of each observation was variable depending on the type of interaction/meeting, ranging from 20 to 210 min. Structured field notes were maintained which included the location and duration of the meeting/interaction, the team members involved, the type of meeting, what was being discussed and by whom, interactions with external agencies and how information about falls was communicated. No resident interactions were observed. Brief short-hand notes were written in a small notebook at the time of the observation to capture key interactions with detailed field notes written immediately afterwards using a structured template (Supplementary information File 2). The structured observation template was created based on the study aims and discussions with the study collaborator group about their experiences of falls management in care homes.

**Table 1:** Types of staff observed

Care home 1	Care home 2	Care home 3
Management team: • Manager • Deputy manager • Clinical leads • Administrative lead	Management team: • Manager • Deputy manager	Management team: • Manager • Deputy manager
Registered nurses	Registered nurses and nursing associates	Registered nurses
Care team: • Senior carers • Carers	Care team: • Senior carers • Carers	Care team: • Senior carers • Carers
Auxiliary teams: • Housekeeping • Kitchen • Maintenance		Auxiliary teams: • Housekeeping • Kitchen • Maintenance • Activity coordinator

Prior to the observations, all care home staff, residents, relatives and external multidisciplinary team members visiting the homes on a regular basis were provided with an information sheet. A researcher attended care home meetings to introduce themselves and explain the study. Staff, residents and relatives were informed that the researcher would make written notes about the organisational structure, communication between different members of the care home team, meeting structures and interaction with other services and relatives but would not record any personal identifiable information about staff or residents. Staff who agreed to the observation taking place were asked to provide written consent. Residents and/or relatives were asked to inform the care home manager if they did not agree for the researchers to be party to any discussions about them/their care. External multidisciplinary (MDT) members were asked to provide verbal consent which was documented by the researcher.

#### Semi-structured interviews

17 semi-structured interviews were undertaken with care staff, commissioners (people responsible for planning and purchasing care for a local organisation) and individuals involved in the organisation of falls management, care home managers, care homeowners and residents (See Table 2). The interviews explored stakeholders’ views on delivering or experiencing falls management in care homes and the challenges and facilitators. The interview schedule presented in supplementary information file 3 was used to ensure a consistent approach. All interviews with healthcare and care home staff were completed by one researcher (KR) and the resident interview was completed by another researcher (FHB). Each interview lasted up to 60 min and took place in person or via phone. Interviews were digitally recorded and transcribed by a researcher or approved transcription company, or notes were made by the researcher, depending on the participant’s preference. Where supporting data is from researcher notes this has been highlighted in the results section. Written informed consent or verbal consent was completed by participants prior to interviews. Participants were offered a £20 gift voucher as remuneration for their time.

**Table 2:** Interview participants

Organisation	Role (number of participants)
Observation care home 1	Care home manager and owner (1) Carer (3) Clinical lead (2) Housekeeper (2) Registered nurse (1) Administrative lead (1)
Observation care home 2	Care home manager and registered nurse (1) Carer (1)
Observation care home 3	Resident (1)
Integrated Care Board	Commissioner (2)
Local Authority	Commissioner (1)
NHS Trust	Falls lead (1)

### Data analysis

**Fig. 1:**
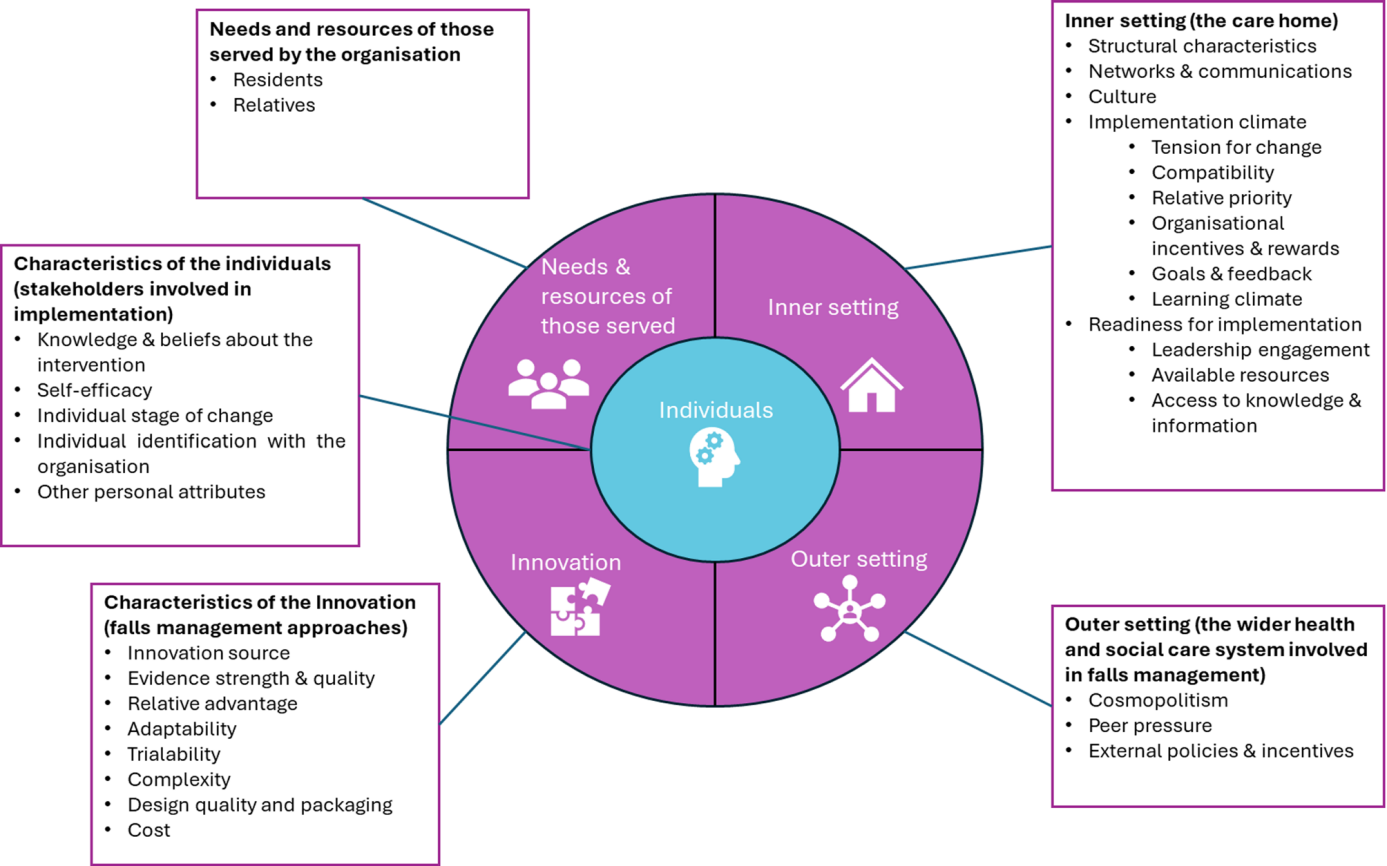
Domains and constructs of the CFIR used in thematic analysis

Thematic analysis was completed using data from the observations and interviews. Field notes, interview transcripts and researcher interview notes were managed using NVIVO 12. The Consolidated Framework for Implementation Research (CFIR) [17] was applied to identify barriers and facilitators to delivering falls management. The CFIR is a recognised implementation framework that been utilised to aid understanding of complex, intertwined contextual factors influencing implementation in health and social care systems [9]. CFIR constructs and domains were used as a coding framework in the analysis to identify factors relating to innovation (falls management), individuals (stakeholders involved in implementation), needs and resources of those served by the organisation (residents and relatives), the inner setting (care home) and the outer setting of falls management delivery (the wider health and social care system) (see Fig. 1). We made two adaptations to the CFIR to align the analysis framework with our research aim: (1) ‘needs and resources of those served by the organisation’ was made a domain rather than a construct in the outer setting; (2) the ‘Implementation process’ domain was removed as the study did not seek to investigate the implementation of a specific falls management intervention, for example a falls management exercise programme. Our coding framework included five domains with 23 constructs as defined in the published 2009 CFIR codebook [17]. Domains and constructs were operationalised through discussion and regular review at analysis meetings with a researcher with expertise in implementation science (ST) to ensure a consistent and rigorous approach.

Data analysis was an iterative process and included the following stages of thematic analysis [18].

1. Familiarisation of the data by reading the data set and noting initial thoughts. Notes on interpretation of the findings were also made at the time of data collection in a reflective journal.
2. Line-by-line coding by one researcher to code the data under the domains of the CFIR (FHB);
3. Codes were then grouped together to generate initial themes under the CFIR constructs (FHB);
4. Themes were sense checked by a second researcher (KR).
5. Themes were then named and described (FHB).
6. Following these stages, themes under the CFIR constructs were mapped by one researcher (KR) to a context coding framework to aid interpretation of the data [19]. The mapped themes were sense-checked by a second researcher (FHB).

An extract of the analysis is provided in supplementary information file 4.

### Reflexivity

The researchers had previous experience of supporting older people working as physiotherapists and acknowledged this in the collection and analysis of the data. Regular reflection was undertaken by journalling and monthly team analysis meetings.

### Patient and public involvement

A collaborator group supported the work and contributed to decision-making about the study design. Group members were engaged through the research team’s existing contacts and networks. The group met online every 3–7 months throughout the work and included a care home quality and policy lead, a PPIE representative with past experience as a GP, carer and care home resident, a consultant therapist in falls prevention, a care home support service matron and a community falls prevention clinical practitioner. The group supported the research design. For instance, the group informed decisions about observation consent processes and offering vouchers as incentives. The group supported the interpretation of the data and dissemination of the findings through discussion of their thoughts on key themes. Members of this group are also co-authors on the manuscript where authorship requirements were met. The PPIE representative was offered remuneration for their time.

## Results

The results were structured with four levels of organisational context: (1) Health and Social Care System; (2) Care home system; (3) Care home team and (4) Individuals. A summary of each of these levels will now be presented.

### Health and social care level determinants

Contextual factors at the wider health and social care level were mapped to six of the CFIR constructs and these are summarised in Table 3.

**Table 3:** Summary of health and social care level determinants

CFIR Domain & Construct	Summary
Outer setting: External Policy and Incentives	**Integrating a disjointed system**: Challenges with integration and collaboration across sectors and effects of separate external commissioning models. Challenges with integration leads to challenges for falls management such as data sharing and duplication.
Characteristics of the innovation: Relative advantage	**Proactive versus inevitability**: Tension between proactively preventing falls and the perception that some falls are inevitable. Apathy could lead to complacency and reduce reporting of the problem contributing to falls risk. Health and Social leaders highlighted the need for a more proactive approach but acknowledged this was challenging to evidence and required culture change.
Characteristics of the innovation: Cost	**Financial pressures**: Financial pressures on the system create challenges to investing in consistent provision specifically in care homes (e.g. specialist falls input, training outside remit, long term implementation of pilot schemes)
Outer setting: Cosmopolitism	**Variation in support available**: Heterogeneity of services available to provide support leads to variation in delivery of falls management practice and confusion over support and access. Attitudes towards care homes affect recruitment to care home support roles, care home staff and health leaders’ experiences of preconceptions about care homes or older people viewed as influencing provision
Outer setting: Cosmopolitism	**Relationships across the system**: Having a consistent and named healthcare professional helped with managing complex needs and access to wider support (e.g. weekly ward rounds). Regular and direct contact was considered important by leaders and managers. Multi-disciplinary decision-making was dependent on care home staff knowledge of residents. Valuing each other’s perspective and feeling heard was considered important but challenging across the system.
Characteristics of the innovation: Complexity	**Complexity of resident’s needs**: Residents often have complex needs. Staff experienced difficulties assessing for and balancing multiple falls risk and collective needs of individuals (particularly residents with dementia). Managing falls is also complex involving a number of interacting factors *e.g. (diet, medication, cognition) and variations in support needed (e.g. general reviews, specific reviews, hospital admissions, equipment).

#### Integrating a disjointed system

There was a recurrent theme that more work was required to break down traditional silos between sectors involved in falls management. Health leaders reported that approaches to address silo working, such as greater care home representation on system wide falls in care homes groups, were a work in progress. NHS staff and care home managers were frustrated at the speed of change. Challenges regarding who should pay for falls management approaches and the different funding mechanisms were identified. Separate record systems created challenges for sharing information about falls across organisations. This added to the time demands on staff and external MDT members due to duplication of documentation.

*‘I think NHS and [name of Local Authority] are already disjointed so to then get us into a disjointed system is difficult*,* but they do try their best.’* (Interview with care home manager).

#### Proactive versus inevitability

There was tension between being proactive to prevent a fall and the perception that some falls are inevitable. Clinical leads felt that this acceptance of falls sometimes led to reduced reporting of problems contributing to falls, such as agitation. Commissioners advocated for a proactive approach in line with national policy drivers but also suggested that culture change was required for care home to see themselves as *‘a key player in the more proactive agenda.’* (Interview with commissioner)

*‘if a resident’s about to fall*,* that you can’t actually stop it anyway. You can do the prevention*,* you can do the tasks like the cleaning up and things like that*,* but when it actually does come to a fall*,* it is mostly purely accident*,* and you can’t actually stop that.*’ (Interview with carer).

#### Financial pressures

Stretched finances across the health and social care system made consistent approaches to falls management difficult. Decisions by health commissioners about whether to fund falls management appeared variable. Some care homes had access to a specialist falls teams whereas others did not, and waiting times were variable. Provision of falls training in care homes by an NHS organisation had been decommissioned due to cost. Pilots needed to demonstrate impact in order to be rolled out across the system, but this was difficult to evidence due to the heterogeneity of care home providers.

*‘resource and funding*,* anything that is done needs to be rolled out consistently across large number of care homes in the area which costs a lot of money. This is a challenge with the current financial pressures and the need to evidence benefit and good value for money’* (Interview with commissioner (recoding and researcher notes).

‘*Historically when there was a specialist falls service rather than kind of a general service that also saw fallers*,* there was a health promotion specialist that provided training into care homes*,* although that was effectively decommissioned*,* because it’s quite an expensive thing to do’ (Interview with falls lead involved in the organisation of falls)*.

#### Variation in support available

Services available to support falls management differed across care homes. For example, homes in one county had access to a specialist falls team whereas homes in another county did not. There was confusion about referral pathways and some cares homes experienced delays in receiving support from external services. Negative preconceptions about care homes amongst healthcare professionals were thought to impact on recruitment to care home support roles and service provision. Health professionals reported these roles were less attractive for some due to fear, a lack of interest and the complex needs of care home residents.

#### Relationships across the system

Weekly ward rounds and a consistent, named health professional linked to the care home supported care homes with accessing wider support to manage complex needs of residents. Relationships between care home staff and wider teams and system partners were crucial as external professionals relied on staff’s knowledge of residents for decision-making. However, issues arose when care home staff and health professionals felt their perspectives had been overlooked when working together, causing frustration.

*‘There is one GP practice that comes to the care home and usually they have the same GP. Nurse thinks this is helpful*,* multiple GPs make things hard because the residents are so complex’’.* (Observation field note)

#### Complexity of resident’s needs

Care home staff worked with residents who had complex needs. Managing falls was also difficult as it involved considering a wide range of risk factors and possible actions. Challenges with managing the impact of multiple falls risks and health needs of individuals were observed, particularly for residents with dementia. Staff also had to consider the safety of others, including other residents and staff, when making decisions about falls. The high level of support needed by care homes, including general reviews, hospital discharge assessments, problem-specific reviews and equipment support, led to challenges with fitting all requests into weekly rounds.

### Care home organisation

Contextual factors at the care home organisational level were mapped to five of the CFIR constructs and these are summarised in Table 4.

**Table 4:** Summary of care home organisation level determinants

CFIR Domain & Construct	Summary
Inner setting: Structural Characteristics	**Environment**: Design of care homes can pose difficulties with supervision, maximising independence and maintaining dignity when a fall occurs. Care homes are busy with noise, interruptions, space constraints and often limited digital infrastructure.
Inner setting: Culture	**Safety Culture**: Safety was described as a main priority in decision-making about falls management by care home staff. Safety influences decision making about falls management and can lead to risk aversion and mistrust between organisations involved.
Inner setting: Implementation climate- Relative Priority	**Competing Priorities**: Falls management includes a number of priorities which include supervision of residents, attending training and promoting person centred activity. Time and cost to the care home and individuals receiving or delivering the intervention (e.g. training, support networks, exercise programmes) can impede implementation efforts.
Inner setting: Implementation climate - Learning Climate	**Analysis and learning approaches vary**: Locally developed approaches to support analysing and learning with the autonomy to try new approaches influenced by organisational structure (e.g. individually owned). Post-falls analysis variable and management driven with limited reflection time.
Inner setting: Readiness for Implementation - Available Resources	**Workforce challenges**: Staffing levels, high turnover and skill mix impacted on implementation of specific falls prevention actions, as well as the ability of care homes to engage in quality improvement initiatives. Providing adequate supervision to prevent falls and promoting activity noted as particular challenges with the staffing resource available.

#### Environment

The design of some care homes was problematic for falls management, with trade-offs between privacy, supervision and activity promotion. While more secluded areas may be more difficult to supervise, staff reported that open communal areas created challenges for ensuring privacy when a fall occurred. Environmental risk factors were important to both residents and care staff. Noise levels, interruptions and space constraints affected communication in meetings where falls were discussed. Some care homes had limited digital infrastructure which restricted the accessibility of virtual or hybrid meetings. Meetings were observed to be virtual, hybrid and in person.

*‘A lot of the residents can’t get through them doors because they’re too heavy. We’ve got one that likes to go her room early*,* but someone has to take her because she’s going through a fire door so she’s not independent.’*  (Interview with resident).

#### Safety culture

Safety was at the heart of decision-making about falls by care home staff. Balancing safety risks against promotion of independence was complex and staff had different views about how best to manage these situations. Criticism by external organisations was perceived to lead to risk aversion and mistrust between those involved.

*‘First thing is safety then you can encourage independence’.*

(Interview with care home clinical lead- researcher notes)

*‘I think care homes as well are really – have been criticised a lot – and kind of perhaps a little bit risk averse if they’ve been shouted at once by an ambulance for the fact of getting somebody off the floor when they should have done’ (*Interview with *falls lead involved in the organisation of falls*.

#### Competing priorities

Falls management had to be balanced with other responsibilities and included many different approaches which created difficulties for prioritisation. Some care homes more overtly prioritised falls discussions in their meetings than others, for instance including falls as a standing agenda item. Implementation of falls management approaches was influenced by the time and cost commitment required by the care home and individuals delivering or receiving the interventions.

*‘So who is high risk of falls? We will make a group and put them inside the lounge and then one of the staff can stand in the lounge and closely monitor for falls. So not always the four staff can stay on the floor*,* sometimes staff need to help with toileting and pad changing so one staff will stay in the lounge with those at risk of falls and the other staff will help the other residents.’* (Interview with carer).

#### Analysis and learning approaches vary

Care homes developed their own approaches to analysis and learning from falls. Ownership of the care home influenced the manager’s autonomy to try new approaches, with individually owned homes having greater flexibility. Falls analysis was led by care home management teams with top-down oversight and variable input from staff who worked on the floor.

*‘up until recently*,* it was not easy to make changes at an individual care home level. Manager thinks this will improve as the regional manager has just started monthly meetings between managers across the region. This is a forum to share ideas and best practice.*‘.

Observation field note)

*‘I think as a medium sized care home but a single care home*,* we do have that ability to be flexible and dynamic in the way we are talking to people*,* we can suddenly have meetings if we want to and we don’t have to go to head office if we want to buy any equipment or stuff like that.’* (Interview with care home manager).

#### Workforce challenges

Workforce challenges were problematic for implementing falls management approaches and engaging with quality improvement work, including staffing levels, turnover and skill mix. Pay and COVID-19 were thought to have exacerbated workforce challenges. Staff became more task-focused and agency workers with less knowledge of residents were used when staffing resource was limited. Providing supervision and promoting activity were noted as particularly difficult with a limited workforce.

*‘The 1–1 is only in place between 1 and 9 o’clock due to agitation levels and so we need to support around those time. The 1–1 is done by an agency staff who watches all the time to prevent falls. Difficulty doing that outside those times.’* (Interview with carer- researcher notes).

### Care home team

Contextual factors at the care home team level were mapped to four of the CFIR constructs and these are summarised in Table 5.

**Table 5:** Summary of care home team level determinants

CFIR Domain & Construct	Summary
Inner setting: Networks & Communications	**Formal and informal communication**: Formal communication channels through structured meetings (handovers, flash round, team meetings). Falls information shared verbally via key connecting roles in a hierarchy (e.g. clinical staff leading, senior carers). Organisation of meetings with external healthcare professionals (e.g. weekly ward) mainly decided by the healthcare organisations involved. Information relating to falls documented in multiple places.
Inner setting: Culture	**Team cohesion**: Care home staff value being united as a team to prevent falls – team-working seen within sub-teams and across teams. Relational strategies- humour when discussing challenging situations, informal chat at start of meetings. However, some instances where divides or disagreements seen between staff working in different roles or shift patterns
Inner setting: Culture	**Set roles**: Staff work within the boundaries of their role and organisation of falls management is hierarchical. Carers and housekeepers were generally closest to residents to detect change however these staff members not always able to be involved in ongoing discussions due to communication structures. Staff played an advocacy role in supporting communication of residents needs and preferences relating to falls
Needs & Resources of Those Served by the Organization: residents and relatives	**Variable level of involvement of residents and relatives in falls-related decision-making (informed versus involved).** Family often involved more than residents due to cognitive impairments and at specific time points (admission to care home, care reviews, best interests, funding, hospital admission decisions).

#### Formal and informal communication

Care homes used daily handovers as the main structure to formally communicate information about falls. Some homes had additional structures in place such as team meetings with senior carers and management teams, which included more in-depth falls discussions, and flash meetings to coordinate daily key priorities across broader teams, including housekeeping and maintenance. Falls information was also communicated informally between staff. Senior carers and nursing staff (where employed by the home) played a key role in passing information up and down a hierarchical communication chain, including delegation, highlighting key priorities and issues. Written communication approaches varied between care homes. Information was recorded in multiple places and formats, including electronic and paper. Weekly rounds, delivered as part of the EHCH framework, were the main structure for communication with external organisations. Attendance of NHS clinicians and the location of the rounds (for instance in-person at the care home or online) varied. Some rounds were GP-led, while another was led by an Advanced Nurse Practitioner. Care homes had limited input into how these meetings were organised which impacted on workloads.

*‘I would prefer it [weekly round] face-to-face I think. If they came into the home and we sat in a room and you can then go and see the resident. We have it on a Thursday and then on a Friday they come and do all the visits which means it like over 2 days… If they were here they could just walk round and assess them there and then and then it’s all done’* (Interview with care home manager).

#### Team cohesion

Team-working and a shared understanding of falls management was key to a successful approach according to care home staff. There were strong working relationships within and across teams. Informal chat at the start of meetings and humour when discussing challenging situations were commonly used to build and maintain relationships. However, there were occasionally tensions and divides between staff working in different roles or shift patterns.

*‘And so if we spill anything we need to clean it immediately*,* we don’t wait for the housekeepers. Whoever is seeing the spillage needs to clean it immediately. Some will say it is the housekeeper’s responsibility but we don’t want to be like that.’* (Interview with senior carer).

#### Set roles

Staff worked within set roles, with senior, qualified and management staff having greater involvement in decision-making and discussions about falls. Staff working ‘on the floor’, such as carers and housekeepers knew residents well and were able to detect changes that may increase their falls risk. Their role was to escalate this information up the organisational hierarchy. Care home staff had contemporary knowledge about residents needs and preferences about falls. They advocated for residents in meetings with external services.

*‘We can’t really discuss anything like that with the relatives. We get a nurse to talk to them… they need to be informed by the nurse first because if they say*,* “Oh we weren’t told about that” then it gets brought up.’(Interview with carer)*.

#### Variable level of involvement of residents and relatives in falls-related decision-making

The degree to which residents and relatives were involved in or informed of falls-related decision- making varied. Families were often included in conversations more than residents due to cognitive impairment. These conversations occurred at specific time points, such as following a fall or when best interests or funding decisions were required. Although considered beneficial, there appeared to be limited information to inform residents and relatives about falls risks and management strategies.

*‘You get the information there if there’s something new come up… the subject of falls*,* no*,* you just take it for granted that it’s there’* (Interview with resident).

### Individual level

Contextual factors at the individual level were mapped to four of the CFIR constructs and these are summarised in Table 6.

**Table 6:** Summary of the individual level determinants

CFIR Domain & Construct	Summary
Characteristics of individuals: Knowledge & Beliefs about the Innovation	**Individual awareness of risks and actions to reduce falls**: Awareness and focus differ by staff role, Carers focused more on the environment, mobility and footwear, with a professionally registered staff considering broader risks and actions. Supervision and environmental factors most important to resident
Characteristics of individuals: Self-efficacy	**Confidence and trust in abilities**: Some staff lack confidence in their own knowledge about falls management and in making suggestions of how to improve falls management whereas others were more vocal in sharing suggestions.
Characteristics of individuals: Individual Stage of Change	**Experience**: Knowledge of falls and skills in falls management differ and builds over time with practical experience. Experiences of falls can be overwhelming for new starters. There are different levels of experience among care home teams, and this is used as a support structure.
Characteristics of individuals: Other Personal Attributes	**Compassion**: Compassionate, caring, motivated, dedicated staff valued by managers and resident, but level of commitment perceived as variable. Perception of residents being a family and having the ability to look out for each other to support falls prevention

#### Individual awareness of risks and actions to reduce falls

Care staff were more aware of risks and actions relating to direct care tasks, such as environmental hazards, footwear and supervising mobility. Nurses, clinical leads and senior care staff tended to consider broader risks, such as the effect of health conditions and medications. Residents’ awareness related to supervision of mobility and environmental risks such as obstacles.

*‘How can we prevent falls?” is a discussion topic in handover. Care staff responses- clean up spillages*,* footwear*,* make sure there aren’t any trip hazards’’* (Observation field note).

#### Confidence and trust in abilities

Some staff questioned their own ability to detect and respond to falls risks. This led to decisions being deferred to internal and external health professionals. Some carers were more confident than others to share their ideas about actions to support individuals or to improve falls management across the care home.

*‘I think because they’ve not really been very empowered to do those assessments and got the skills that it’s then like ‘oh no someone else has to make this decision about stuff so that needs to be a professional that decides that’’* (Interview with *with falls lead involved in the organisation of fall*).

#### Experience

Staff knowledge and skills in falls management built over time until it became instinct. Witnessing falls and working with residents with complex needs could be overwhelming for staff with limited care experience. More experienced staff helped less experienced staff to develop knowledge and skills by working with them on shifts and acting as a mentor.

*‘So you have those people who have been in care for years and have that experience and they can mentor the other staff. So they know instinctively if someone might be about to have a fall*,* do they have a UTI*,* do they need a med review*,* are they eating enough of their lunch*,* what is their natural behaviour*,* can we try fortified diet? They have all that going on in their heads but you bring in someone brand new to care and they obviously have to develop that over time.’* (Interview with care home manager).

#### Compassion

The compassion and motivation of staff supporting falls management was of high value to managers and residents. However due to multiple competing priorities and complexity it was perceived challenging for staff to consistently champion falls management. The resident interviewed demonstrated compassion towards each other and viewed fellow residents as family. They supported falls management to help protect each other from harm.

*‘I’m more vigilant because I sit there and take it all in*,* I see it all happen and I can sometimes see it before the staff can see it… It’s like a mother instinct… I’ve been here eight years now - and it’s my family’* (Interview with resident).

## Discussion

By observing and talking with care home staff and individuals involved in the organisation and management of falls we have explored the contextual issues of delivering falls management in care homes. The conclusions drawn from this study need to be considered within the context that the data was drawn from a small number of care homes in one region in England and may not be generalisable outside of this region. Barriers included difficulties in integration across a disjointed system, workforce challenges and managing the complexity of resident needs with multiple competing priorities. Facilitators included consistent and regular multi-disciplinary support, valuing team working within the care home, and between the care home and external agencies, and the ability to retain care home staff who developed and honed skills over time and who valued their advocacy role for residents. Variation in care home environments and access to healthcare support were highlighted. The corporate structure of care homes affected approaches to analysis and learning from falls, with individually owned care homes considered to have greater autonomy.

### Strengths and limitations

This qualitative study has explored in depth the context for falls prevention delivery in a small number of care homes in one area of England, all of which were registered for nursing care. We acknowledge that the findings need to be considered within the limitation that the homes observed may not reflect the broad and diverse care home sector and the findings may not be generalisable beyond the region the work has been conducted. Although the region where the work took place had implemented several nationally mandated initiatives – such as the EHCH framework – it is important to recognise that this is also a part of the country with a long history of collaborative working which laid the foundation for locally-led vanguard initiatives in the mid 2010s [13] and a history of wider collaborative working between the care sector, NHS and academics around falls and falls prevention [20]. It is possible that other systems, in other parts of the country, may not have had as long to develop shared ways of working between care homes and the NHS.

The aim of the work was also to explore the contextual factors which may have focused on more challenges and issues rather than facilitators. The findings presented need to be considered as an interpretation of the contextual factors based on a small number of homes and the basis in which to explore further implementation strategies within this context.

In addition, we did not observe any resident interactions and only one resident took part in an interview. Although observation of staff interaction with residents was considered, it was not undertaken due to data protection and confidentiality concerns highlighted during the ethical review process. Other ethnographic studies in health and social care settings have encountered similar challenges following an update of data protection and information governance legislation in the UK [21, 22]. Our findings are therefore limited to the perspectives of care home and NHS staff. A scoping review on the barriers for care homes residents to participate in research highlighted that many factors, such as research methods and communication, are not in the control of residents [23]. The lack of resident voice in this work is recognised as a limitation and further work is needed to explore how to include residents and relatives in the delivery of falls management and falls prevention research.

A strength of this work is that the observations and analysis in this work followed a transparent and structured process with regular reflection, discussion and interpretation of the findings with a study collaborator group. This group included clinicians, care home staff and care home policy managers which helped to interpret the data in the context of the care home community.

### Wider context

The complexity in managing falls for care home residents was highlighted in this study, with a number of interacting components within a complex system. There are challenges to considering individual issues, such as falls, in isolation when considering comprehensive and integrated approaches to care of older people with frailty, including those that live in care homes. Care home residents live with multiple long-term conditions and take multiple medications and treatments for them [24]. Most recommendations to improve healthcare provision to residents focus on increased integration, comprehensive assessment and coordination of care taking account of the diverse multidisciplinary expertise required [25]. It’s important to see falls initiatives as situated in these wider approaches but doing so may further amplify the implementation challenges described here.

The work replicates findings seen across multiple previous studies about the differing perspectives of NHS and care home staff and the need to identify shared priorities and common mutually beneficial goals [26]. A long literature has outlined the counterproductive and alienating effect for care home staff of externally imposed policies and initiatives that demand time and resource, without recognising the associated opportunity costs and pressures [27]. We saw, within our responses, some evidence where work had been undertaken to build relationships with care home staff and to use these as the basis of shared initiatives. There was evidence of the NHS funding specific staff members or teams to support falls management. There were, however, also examples of NHS staff and services to support falls management in care homes that were reconfigured or restructured. In some cases, this led to care home staff feeling they are working in isolation with less support. Previous literature around implementation of healthcare initiatives in care homes has suggested a greater likelihood of success where – in addition to the efforts to build on existing relationships described here – time and money are invested to pay for care home staff time and help them develop skills related to quality improvement and implementation [28]. Interventions will be more likely to embed and sustain in care homes where there is a focus on monitoring outcomes and regular check-in across NHS and care home staff, ongoing communication and awareness raising between these groups, and attention to detail in addressing care home staff and organisational priorities [11]. Whilst we saw evidence in this study that collaborative working was seen as key to success, we will need to explore strategies that focus on collaborative and on-going support in the future implementation of the Action Falls programme. This may be particularly important in managing what happens when a fall does occur as feelings of being shouted at or told off for moving or not moving a resident off the floor were expressed in this study. These interactions were not observed as part of this study and therefore further research is needed to explore the perspectives raised in this study.

Workforce challenges were identified as a barrier to falls management in this study with care staff considering experience in the setting to be important in successful falls management. Retention of care staff across long term care is recognised as a global challenge [29, 30] and a high turnover of staff can lead to poorer quality outcomes of care [31], including falls [32]. Training opportunities for care staff may encourage retention [29] and varied opportunities for training in falls management were discussed by participants in this study. Limitations with training were highlighted given the complexity of resident needs. Practical experience gained by care staff appeared to develop more instinctive skills and confidence in falls management which provided support for staff with more limited experience. To ensure the sustainability of this model of experienced care staff supporting newer colleagues, there is a need to consider the wider context and challenge of workforce retention in the sector.

In view of the contextual factors highlighted in this work, future research needs to work with the care home sector to co-produce strategies that will support the Action Falls programme to be embedded into care homes.

## Conclusion

The delivery of falls management in care homes is complex involving a number of interacting systems, professionals and competing priorities. Designing implementation strategies that embed falls management into care homes requires consideration of pressures on care homes and wider systems, and how established support networks can facilitate delivery and variation between settings.

## Electronic supplementary material

Below is the link to the electronic supplementary material.

Supplementary Material 1

## Data Availability

The data analysis spreadsheet is available on request from the corresponding author. The original field notes and interview transcripts are not for sharing to maintain confidentiality of the participants due to the small number of care homes participating in the observations.
