# Supplementary material for: Exploring the organisation and delivery of falls management in care homes for older people in England
Source: BMC Geriatr. 2025 Jul 1;25:433. doi: 10.1186/s12877-025-06127-w (PMC12219685; doi:10.1186/s12877-025-06127-w)
Supplement: Supplementary file 1 — Supplementary Material 1 [file 12877_2025_6127_MOESM1_ESM.docx]

Supplementary Files

File One: Standards for Reporting Qualitative Research

File Two: Structured field note template

File Three: Interview Schedule

File four: Extract of analysis

File One: Standards for Reporting Qualitative Research

| **Title and abstract** | **Pages** |
| --- | --- |
| **Title** - Concise description of the nature and topic of the study identifying the study as qualitative or indicating the approach (e.g. ethnography, grounded theory) or data collection methods (e.g. interview, focus group) is recommended | 1 |
| **Abstract** - Summary of key elements of the study using the abstract format of the intended publication; typically includes background, purpose, methods, results, and conclusions | 2 |
| **Introduction** | |
| **Problem formulation** - Description and significance of the problem/phenomenon studied; review of relevant theory and empirical work; problem statement | 3 |
| **Purpose or research question** - Purpose of the study and specific objectives or questions | 3 |
| **Methods** | |
| **Qualitative approach and research paradigm** - Qualitative approach (e.g., ethnography, grounded theory, case study, phenomenology, narrative research) and guiding theory if appropriate; identifying the research paradigm (e.g., postpositivist, constructivist/ interpretivist) is also recommended; rationale | 3 |
| **Researcher characteristics and reflexivity** - Researchers’ characteristics that may influence the research, including personal attributes, qualifications/experience, relationship with participants, assumptions, and/or presuppositions; potential or actual interaction between researchers’ characteristics and the research questions, approach, methods, results, and/or transferability | 5 |
| **Context** - Setting/site and salient contextual factors; rationale | 3-5 |
| **Sampling strategy** - How and why research participants, documents, or events were selected; criteria for deciding when no further sampling was necessary (e.g., sampling saturation); rationale | 3-5 |
| **Ethical issues pertaining to human subjects** - Documentation of approval by an appropriate ethics review board and participant consent, or explanation for lack thereof; other confidentiality and data security issues | 14, 17 |
| **Data collection methods** - Types of data collected; details of data collection procedures including (as appropriate) start and stop dates of data collection and analysis, iterative process, triangulation of sources/methods, and modification of procedures in response to evolving study findings; rationale | 3-5 |
| **Data collection instruments and technologies** - Description of instruments (e.g., interview guides, questionnaires) and devices (e.g., audio recorders) used for data collection; if/how the instrument(s) changed over the course of the study | 3-5 |
| **Units of study** - Number and relevant characteristics of participants, documents, or events included in the study; level of participation (could be reported in results) | 3-5 |
| **Data processing** - Methods for processing data prior to and during analysis, including transcription, data entry, data management and security, verification of data integrity, data coding, and anonymization/de-identification of excerpts | 3-5 |
| **Data analysis** - Process by which inferences, themes, etc., were identified and developed, including the researchers involved in data analysis; usually references a specific paradigm or approach; rationale | 5 |
| **Techniques to enhance trustworthiness** - Techniques to enhance trustworthiness and credibility of data analysis (e.g., member checking, audit trail, triangulation); rationale | 3-5 |
| **Results** |  |
| **Synthesis and interpretation** - Main findings (e.g., interpretations, inferences, and themes); might include development of a theory or model, or integration with prior research or theory | 6-13 |
| **Links to empirical data** - Evidence (e.g., quotes, field notes, text excerpts, photographs) to substantiate analytic findings | 7-13 |
| **Discussion** |  |
| **Integration with prior work, implications, transferability, and contribution(s) to the field** - Short summary of main findings; explanation of how findings and conclusions connect to, support, elaborate on, or challenge conclusions of earlier scholarship; discussion of scope of application/generalizability; identification of unique contribution(s) to scholarship in a discipline or field | 13-15 |
| **Limitations** - Trustworthiness and limitations of findings | 13 |
| **Other** |  |
| **Conflicts of interest** - Potential sources of influence or perceived influence on study conduct and conclusions; how these were managed | 17 |
| **Funding** - Sources of funding and other support; role of funders in data collection, interpretation, and reporting | 17 |

File Two: Structured field note template

| Observer:  Date and time:  Site:  Staff consent log completed: YES/NO  Resident opt-out check completed: YES/NO | |
| --- | --- |
| **Role of people involved** *E.g. carer, CH nurse, CH manager, GP, health care professional* | **Reflections** |
| **Meeting/interaction** *Location, duration, structure* |  |
| **Information shared** *Details of a fall or actions to manage falls, written or verbal* |  |
| **Communication** *Who does/doesn’t speak? Who leads the conversation? Who makes decisions?* |  |

File Three: Interview Schedule

**Interview Schedule: Residents**

**Schedule**

Up to 25 interviews will be conducted with a range of stakeholders involved in falls management in care homes. These will include care home residents. The interview will be semi-structured and will allow for participants to explore new areas as the interviews develop. Interviews will be managed to last no longer than one hour.

**Topic Areas**

The interviews will be semi-structured in nature to allow exploration of key areas relating to the delivery of falls management as well as explore new areas identified by participants. An outline of the key areas to be explored and example questions are provided below. The areas will be further informed by the observations in component one. The exact nature and wording of the questions will vary to allow the interview to be guided by the participant and explore areas important to them. The questions will be appropriately worded to reflect the stakeholder group of the participant.

**Introductory Interview Script:**

“Thank you for agreeing to take part in the interview today to talk about your experiences of falls and how staff support you in thinking about your potential risk of falls.

Taking part in the interview is completely voluntary. There are no right or wrong answers, we are interested in your views and experiences of reducing your risk of falling and how this has been done within your care home.

We anticipate that the interview will last no longer than an hour but please stop me at any point if you would like a break or would like to stop the interview.

We will [*record the interview with your consent/make written notes along the way*] and with your permission anonymous quotes may be used in reports to share the study findings.

To maintain confidentiality, we ask that you please do not mention any personal information or names that may identify specific staff or other residents.

The content of the interview will remain confidential and only used for the purpose of this research however if we consider anything you disclose during the interview requires escalation as it relates to harm of another person we will be obliged under our professional code of conduct to follow safeguarding procedures of our employer Nottingham University Hospitals NHS Trust.

We do not anticipate that the interview will be distressing, but please only answer and talk about things that you feel comfortable with. If you do feel uncomfortable at any time please just let us know and we can stop the interview at any point. If you would like to have someone you know well (for example a relative or a friend) with you during the interview process please just let us know.

Is there anything you would like to ask us at this point before we start the interview? Are you happy to continue with the interview?”

**Introductory Questions**

The participant’s response should be present on the recording, or documented in the notes. If the participant confirms they are happy to proceed with the interview, this will start with some broad questions to introduce the interview and encourage the participant to discuss their view. These questions will include:

- Can you tell me about a time when falls were discussed with you?/ Can you tell me about a time when you have experienced of a fall in the care home?
- Please talk about how you feel about the support that you are given in the care home to prevent you from falling/please talk about how you feel about falling?

**Key Themes**

The interview will focus on the key areas of interest below:

- **How is information about falls shared with you?**
- **How are you are involved in discussions and decisions about falls?**
- **How do care staff and health professionals (e.g. your GP/physio) involved in your care, work together?**
- **What is good about how falls are reduced or handled in your care home?**
- **What makes it difficult to stop or reduce falls in the care home?**
- **How could things be done differently?**
- **What could help to support you/other residents to be involved in decisions about how falls are managed in the care homes?**
- **What skills and knowledge do you/other residents have that could help prevent or manage falls?**
- **What other skills and knowledge would be useful?**

**Interview Schedule: Staff**

**Schedule**

Up to 25 interviews will be conducted with a range of stakeholders involved in the delivery of falls management in care homes. These will include care home staff, care home managers, care home training leads, commissioners and care home residents. The interview will be semi-structured and will allow for participants to explore new areas as the interviews develop. Interviews will be managed to last no longer than one hour.

**Topic Areas**

The interviews will be semi-structured in nature to allow exploration of key areas relating to the delivery of falls management as well as explore new areas identified by participants. An outline of the key areas to be explored and example questions are provided below. The areas will be further informed by the observations in component one. The exact nature and wording of the questions will vary to allow the interview to be guided by the participant and explore areas important to them. The questions will be appropriately worded to reflect the stakeholder group of the participant.

**Introductory Interview Script:**

“Thank you for agreeing to take part in the interview today to talk about your experiences of managing falls as a member of staff working in a care home*.*

Taking part in the interview is completely voluntary. There are no right or wrong answers, we are interested in your views and experiences of reducing the risk of falling and how this has been done within your care home.

We anticipate that the interview will last no longer than an hour but please stop me at any point if you would like a break or would like to stop the interview.

We will *[record the interview with your consent/make written notes along the way]* and with your permission anonymous quotes may be used in reports to share the study findings.

To maintain confidentiality, we ask that you please do not mention any personal information or names that may identify specific staff or residents.

The content of the interview will remain confidential and only used for the purpose of this research however if we consider anything you disclose during the interview requires escalation as it relates to harm of another person, we will be obliged under our professional code of conduct to follow the safeguarding procedures of our employer Nottingham University Hospitals NHS Trust.

We do not anticipate that the interview will be distressing, but please only answer and talk about things that you feel comfortable with. If you do feel uncomfortable at any time, please just let us know and we can stop the interview at any point.

Is there anything you would like to ask us at this point before we start the interview?

Are you happy to continue with the interview?”

**Introductory Questions**

The participant’s response should be present on the recording, or documented in the notes. If the participant confirms they are happy to proceed with the interview, this will start with some broad questions to introduce the interview and encourage the participant to discuss their view. These questions will include:

- Can you tell me about your role in supporting falls prevention in care homes?
- Please talk about how you feel about trying to support falls management programmes/please talk about how you feel about falling?

**Key Themes**

The interview will focus on the key areas of interest below:

- **How are falls programmes currently delivered?**
- **How do you collaborate across different service and organistions?**
- **What are the key challenges to delivering falls management in care homes?**
- **What are the key facilitators to delivering falls management in care homes?**
- **How would you organize things differently?**
- **What could help to support you in delivering falls management programmes?**
- **What skills and knowledge do you have for supporting falls management?**
- **What other skills and knowledge would be beneficial?**

File Four: Extract of analysis

| **Domain** | **Construct** | **CFIR Definition** | **Theme** | **Example of supporting data (Quotes from interviews or excerpts from field notes)** |
| --- | --- | --- | --- | --- |
| Innovation characteristics | Innovation source | Perception of key stakeholders about whether the falls management intervention is externally or internally developed. | **Credible source** Falls training and information should be from a credible source but views are mixed on who is perceived as a credible expert. Some care home staff information should be provided by professionals external to the care home with specialist knowledge, whereas other care home staff and health and social care leads felt that training should be led by someone within the care sector expertise to incorporate their practical, real-world knowledge. Some felt that trainer's knowledge and experience, and its relevance to their residents and care home, was more important than whether they were internal or external to the organisation. One manager suggested involvement of industry leaders or care homes with outstanding CQC ratings would add credibility to training, whereas a resident felt that university hospital sources of information would be more influential. | ' I could be interested in something like that. To read more... from the professional’s point of view. You’ve got it from, er, a person’s, a falls specific falls person, so a professional person, you know, that might be beneficial to me.'  'for people to take notice it’s got to come from an organisation such as the university hospital. Now when I see that university hospital I know it’s a teaching hospital. It’s coming from a teaching hospital and a group of physios or whatever, and people might take notice' (RES001)    'for me' that person would have more credibility if that person was involved in the care home industry.' (CHS002)  'I: And would that be better if it was someone who had worked in a care home, or a professional who has come into a care home? R: Erm I suppose either. I suppose even if it’s someone even observing or auditing it can help. From a care home point of view if they have come from a residential care home it is completely different from what we are dealing with so they might tell us things that aren’t things that we can actually achieve here. So I think some knowledge of dementia would help.' (CHS010)   'The best training in-house from someone with knowledge and knows what they are dealing with...If someone turned it to do training I would just assume that they knew what they were doing and talking about- why would they be there and turn up if not! I would be surprised if they didn’t know what they were doing- why would they do it? It could be someone from the NHS, local authority it wouldn’t matter' (CHS011) |
| Innovation characteristics | Relative advantage | Stakeholders’ perception of the advantage of implementing the intervention versus an alternative solution. | **Falls Training** Falls training may beneficial but is not seen as a substitute for on the job experience and needs to have tangible benefits from manager perspective. Differences in opinion about whether residents and relatives should or would be able to take part in training but there was a common view among care home staff was that this may help with understanding between relatives and staff. Staff felt relatives were unaware of why some actions were necessary to reduce falls risks or lacked confidence in supporting falls management. HSC view that a standardised system wide training offer would be beneficial. | what I find is, it take us about an hour and half to do your training and you get a certificate but we use a guinea pig. That guinea pig might be me one day, might be one of the other trainers you know. I am not a dementia patient…I can act but I am not that good [laughing]. Erm until they are actually out on the floor they don’t see how a dementia patient behaviours with a fall. Erm but sometimes you get that shock horror type.. because its so easy when you are in a room and you have that type of equipment, its so easy.' (CHS005)  'The outcome to the residents... so say we have had 60 falls or over the last four months we have had an average of 50 falls let’s say and then we did the training and then following this training the next four months we had 25. Then I would say yeah can’t argue with that we have got to take part.' (CHS002)   Some residents might be able to participate – depends really.' (CHS012)   'I don’t think any of our residents would be able to.' (CHS001)  'In terms of looking at a system approach to offering training, its not just for care homes its at all levels from carer level, family level right up to consultant, healthcare professional level and that would include care home staff. I think that could be a real trail blazing model where we have got system wide agreement that there is a standardised training offer that you could access depending on what your role was. I think moving forward that will be a really positive model.' (HSC001) |
|  | Relative advantage | As above | **Proactive versus inevitability** Sometimes a proactive approach was advocated to prevent falls but some falls were perceived as inevitable. Apathy could lead to complacency and reduce the reporting of the problem. Proactive action commonly involved monitoring and screening for infections, using sensor mats to alert staff when a resident was mobilising without supervision and removing environmental hazards. HSC leaders believed that a more proactive approach was needed to address falls in care homes but acknowledged that achieving this was challenging to evidence and required culture change. | No, I don’t think there is anything because you can’t really learn things like that because if a resident’s about to fall, that you can’t actually stop it anyway. You can do the prevention, you can do the tasks like the cleaning up and things like that, but when it actually does come to a fall, it is mostly purely accident, and you can’t actually stop that.' (CHS003)  'I think it would be really helpful for the care homes to understand how key their role is in preventing falls and that it doesn’t have to be inevitable. I mean obviously you can’t stop every fall and with frailty and to a degree not every fall can prevented. But I think there can be bit of a culture of you know we have got some many fallers as a collective term. And how do we empower care staff to really think I am a key player in the more proactive agenda. I don’t know what the answer is these things are easy to say but harder to do aren’t they?' (HSC001) |
| Innovation characteristics | Complexity | Perceived difficulty of the intervention, reflected by duration, scope, radicalness, disruptiveness, centrality, and intricacy and number of steps required to implement. | **Complexity of residents' needs** Residents often have complex needs. Staff experience difficulties assessing for and balancing multiple falls risk and health needs of individuals (particularly residents with dementia). Consideration of the safety of others (staff, residents) was also a factor in decision making. Conflicting view about walking aid use being 'common-sense' from HSC leader in comparison with the view care home staff that implementation of walking aid was challenging for some individuals. | 'Aggression towards staff and giving medication before personal care discussed. Clinical lead explains this is a balancing act due to high risk of falls.' (Field note CH001 02.08.22)  'I think here if someone does have a fall it can be hard to try and make sure they don’t cause anyone else to fall.' (CHS010)  'Not all of it is but I think there’s certain bits and you do just kind of go like ‘well is it not common sense that if someone’s unstable when they're walking that they need to use a walking frame’?' (HSC002)  'Well I think everyone is different, aren’t they? Can’t treat everyone the same can you and everyone is different. You know I have some residents who have walking aids that won’t use them, some that leave them anywhere…(CHS009) |
| Innovation characteristics | Design Quality & Packaging | Perceived excellence in how the intervention is bundled, presented, and assembled. | **Alternative approaches to how falls management messaging** is packaged suggested by HSC leaders such as bringing in different expertise and a more consistent approach across the system. | It would also be useful to have more skills in communicating and engaging with different stakeholders- should we be targeting care home residents with different information than staff? How is the best way to communicate and get messages across in a meaningful way? We often hear care home staff aren’t going to have time to sit and read e-mails and newsletters so how can we market the information appropriately? Comms and marketing skills and training would be useful.' (HSC004)   'So public health, it is making it public health. So how do we get care home staff to think not just about falls but making every contact with falls. So it is whether falls is too niche, do you get what I am saying. I sometimes perhaps think perhaps it is too niche and we have an umbrella where falls sits underneath it and you are going to get your gains from it more than just falls. Equally it can be education with care home staff and getting them to understand not just the impact of falls but the impact of healthy initiatives and they all holistically join up and make every contact count. So falls could be one part of that but one of 10-15 interventions so tobacco. So that could be a way. But equally at the same time falls are huge' (HSC003) |
| Innovation characteristics | Cost | Costs of the intervention and costs associated with implementing the intervention including investment, supply, and opportunity costs. | **Time and cost of falls approaches**   Inner setting- time and cost to the care home and individuals receiving or delivering the intervention ( e.g. training, support networks, exercise programmes) can impede implementation efforts Outer setting-Cost of falls acknowledged but pressures on the system create challenges to investing in provision specifically in care homes and decommissioning (e.g. specialist falls input, training outside remit) . Financial constraints were described as a challenge to investing in consistent falls management provision in care homes across the ICS. Financial challenges wider than falls were also highlighted by HSC leaders (requirements of evidencing benefits to support roll out of improvement pilots creating greater initial focus on larger or higher performing care homes where ‘you get your gains’, challenges of separating costs across health and social care) | some of the things that [researcher name] has brought to use such as the care manager network that would be a good thing to get involved in and then it is just getting people in to have extra training can be a challenge with staffing that is sufficient and at capacity. Erm so we would always like to have more training, it motivates people. We would like to have more specialist training but it can be a challenge to get that event if it is provided free of charge... in an ideal world if you had a training tsar, a regional training tsar that said this is the top-notch best training you guys can have in falls and we want you to have it and its free. That would be perfect.... maybe some people would do it, some carers might do it, if it was something in it for them. I mean it has to be quid pro quo as I mean you are taking time out of their family lives you know where they could be spending time with kids you know.' (CHS002)  'You know they were waiting to do their functional assessment erm, and after a bit it is quite difficult as I say you have a group of 10, not even 10, more than 5 of residents waiting patiently and to do them properly they take 3-4 minutes, so when there is 5- 10 residents you are eating into half an hour of the first session and it quite easy for the residents, some of them anyway, to become disillusioned thinking what am I doing waiting for all this to happen and then only having 15-20 minutes of exercise.' (HSC003)  'Well historically [name of area] you can tell that resources are being pulled out of [name of area]... so falls they had someone in post and then I think they left so not as much with the falls. But yes there has been in the past specialist teams coming in on tap and that was great and I think that is slightly shifting now and rebalancing and looking at different ways of doing things?' (CHS002)  'as I say from my knowledge it can be that I know with certain interventions, not necessarily pilot projects relating to falls, I am not talking about them...there is always that focus on you go to those bigger homes and you get your gains and you go yay. And I really do think it is challenging in care homes as I just think they are really hard, there are so many differences. So you could use say premier league, top performing, good finances and part of a big chain. I am not saying they do well all the time but they have that funding model and they have that sustainability to buy bigger and get the sort of better staff and retain the staff and get that nice culture in that care home. Where we need to be looking at a lot of those smaller care homes and focus on those' (HSC003)  'resource and funding, anything that is done needs to be rolled out consistently across large number of care homes in the area which costs a lot of money. This is a challenge with the current financial pressures and the need to evidence benefit and good value for money' (HSC004)  'I did a training thing the other day on kind of integrated working and it was kind of, like, looking at well you’ve got the Care Act and you’ve got the NHS Act and how are things funded and how we do work together. And it does feel like a real minefield And I kind of think tend to kind of fall more under health I think because we think about people’s mobility and know their medical conditions and their medications and all those kind of things. I think there’s an awful lot of stuff that would also fit under social services type umbrella... and then the cost of falls often falls under social services in terms of well this person now needs one to ones or they need a nursing home placement rather than, you know, a higher level of care or whatever. So the cost burden often falls under social services, so whether you can go well actually if we did this better' (HSC002) |
